# A Review of Our Knowledge of Phyto-Bezoars

**Published:** 1898-03

**Authors:** J. W. Harshberger

**Affiliations:** Instructor in Botany, Veterinary Department, University of Pennsylvania


### A REVIEW OF OUR KNOWLEDGE OF PHYTO-BEZOARS.

By J. W. Harshberger, Ph.D.,
INSTRUCTOR IN BOTANY,
VETERINARY DEPARTMENT, UNIVERSITY OF PENNSYLVANIA.

Considerable interest has been aroused among veterinarians, farmers, and stock-raisers in the United States as to the injury caused to domestic animals, especially horses, by their eating too freely of certain kinds of food. For some time it has been known that cattle turned into rich clover-fields had become sick by feeding too greedily upon the rich and succulent clover-tops. Bloating has been caused by the too free use of such plants.

It is not, however, to this phase of the subject that I wish to revert. A new forage-plant has been recently introduced into America, and has met with much favor among farmers, because it has been found to be quite a good soil-renovator, and yields an abundant and nutritious crop of fine forage. Unfortunately, however, the plant has fallen into partial disrepute, owing to the injurious effects of the hay which was cured after the plant had begun to flower. Dr. F. V. Coville, Botanist of the Department of Agriculture at Washington, has given us an account of a few cases.[1] Mr. William P. Corsa forwarded to the Department of Agriculture a ball of peculiar appearance, stating that it had been taken from the stomach of a horse belonging to Joseph W. Messick, of Milford,

[1] Coville: Crimson Clover Hair-balls. Circular No. 8, Div. of Botany, United States Department of Agriculture, June 15, 1895.

Del., which had been eating crimson clover (*Trifolium incarnatum*), and the death of which was ascribed to the ball. The statement was also made that Mr. Alexander Ryan a few days before had lost a horse from which two similar balls were taken. "About the same time another letter, from an entirely different locality, Kellar, Va., written by B. W. Mears & Son, was received by the Department, accompanied by a ball taken from the stomach of a horse immediately after death. The statement was made that the horse had worked as usual without any signs of disease up to the time of its fatal illness. The horse was suddenly taken with intense pain, and lived only five hours. Another ball, similar to that taken from the stomach was found in the large intestine. Several other horses in the vicinity had died the preceding week, all apparently from the same cause, and the farmers had ascribed it to the feeding of crimson clover." Another case was reported in the summer of 1895 by Dr. Charles F. Dawson, of Washington, who received from a veterinary surgeon of Raleigh, N. C., three balls which he had removed from the intestine of a horse after death.

The balls received were nearly uniform in appearance. They were almost spherical, and varied from three to four and one-half inches in diameter. A microscopic investigation revealed the fact that they consisted of minute, rather stiff hairs, seldom more than one-tenth of an inch in length, sharply pointed at one end, and enlarged or broken off at the other. A comparison of these hairs with those found on the mature crimson clover showed the two to be identical. They become stiff when the plant matures, but in the immature plant are soft and flexible. If horses feed upon overripe crimson clover, the bristly hairs will accumulate and cause death by the formation of one or several of these phyto-bezoars.

Prof. William Trelease, Director of the Missouri Botanical Garden, reports[1] a still more interesting phyto-bezoar. "In January, 1897, Dr. Francis Eschanzier, of San Luis Potosi, Mexico, sent to me two specimens, one a ball of surprising accuracy of surface, measuring a little over three and one-half inches in diameter, and weighing seven and one-half ounces, and the other, one-half of a similar ball, about four inches in diameter, and weighing four ounces, stating that sixteen such balls had been taken from the stomach of a bull at the Hacienda de Cruzes, and adding that he believed them to be composed entirely of an agglomeration of the fibres of some cacti, an undigested portion of which formed the

[1] Trelease: Transactions of the Academy of Science of St. Louis, vii. p. 493, November 30, 1897.

nucleus." Inspection proved this supposition to have been a correct one. The specimens were of a brown color, and consisted of the barbed hairs with which the pulvini of the *Platopuntias* are armed. The cattle turned loose in the field are allowed to eat the cacti, as they are quite nutritious, and so the barbed hairs had accumulated in ball-like form until death resulted.

Balls formed of hairs are likewise found in the stomachs of ruminants, to which they have found their way when the animals have licked themselves. Such a one, preserved in the Museum of the Iowa Agricultural College, weighs four pounds and eleven ounces, and measures twenty-five and one-half inches in circumference.[1]

Occasionally concretions of similar form appear in the human stomach. In 1894 Dr. W. B. Outten[2] found one of a conical shape, five inches in diameter in its broadest portion, and weighing fourteen ounces. "These masses appeared to have been formed about a quantity of vegetable connective tissue, intermingled with starch, etc., and subsequently increased by the same materials, yeast-cells, etc.

"In discussing the use of oat-bran as a food for domestic animals, especially horses and donkeys, Dr. Harz[3] characterizes it as a dangerous food-material, because it favors the formation of large bezoars, which he had previously discussed in an extensive paper,[4] in which is given a classification of structures of this kind, with a very considerable citation of earlier literature."[5]

In the West and Southwest, where one of the opuntias with longer spines is fed to cattle (*Opuntia Engelmanni*), it is customary to remove the long spines, but this does not entirely remove the danger of their use. The late Dr. Vasey, Botanist of the Department of Agriculture, in a publication of that Department gives a number of instances in which cattle have died from an accumulation of spines in the mouth and stomach.

This review of our knowledge of phyto-bezoars clearly shows that the subject of disease as caused by plant-foods is an important one for veterinarians, farmers, and stock-raisers to comprehend, because upon it depends the safety of cattle that are turned out without supervision into the open pasture.

1 Loc. cit.

2 "A Case of Double Gastrolith Removed by Gastrotomy." The Medical Fortnightly, St. Louis, August 15, 1894.

3 Harz: Landwirthschaftliche Samenkunde, ii., 1315.

4 Beiträge zur Kenntniss der Pflanzenbezoare des Pferdes und Rindes Deutsche Zeitschrift für Thier medicin und vergleichende Pathologie, i. 393–407, 1875.

5 Trelease: Loc. cit.

